# Clinical Relevance of the Anti-inflammatory Effects of Roflumilast on Human Bronchus: Potentiation by a Long-Acting Beta-2-Agonist

**DOI:** 10.3389/fphar.2020.598702

**Published:** 2020-12-08

**Authors:** Hélène Salvator, Amparo Buenestado, Marion Brollo, Emmanuel Naline, Tatiana Victoni, Elisabeth Longchamp, Hermann Tenor, Stanislas Grassin-Delyle, Philippe Devillier

**Affiliations:** ^1^ Laboratory of Research in Respiratory Pharmacology, V2I - UMR-0092, Université Paris Saclay, Suresnes, France; ^2^ Department of Airway Diseases, Hôpital Foch, Suresnes, France; ^3^ Department of Pathology, Hôpital Foch, Suresnes, France; ^4^ TOPADUR Pharma AG, Schlieren, Switzerland; ^5^ INSERM U1173, Infection and Inflammation, Département de Biotechnologie de la Santé, Université Paris-Saclay, Montigny-le-Bretonneux, France

**Keywords:** roflumilast, beta-2-adrenoceptor agonist, human bronchus, cytokine, lipopolysaccharide

## Abstract

**Background:** Roflumilast is an option for treating patients with severe COPD and frequent exacerbations despite optimal therapy with inhaled drugs. The present study focused on whether the phosphodiesterase (PDE) 4 inhibitor roflumilast and its active metabolite roflumilast N-oxide affect the release of tumor necrosis factor (TNF)-α and chemokines by lipopolysaccharide (LPS)-stimulated human bronchial explants. We also investigated the interactions between roflumilast, roflumilast N-oxide and the β_2_-agonist formoterol with regard to cytokine release by the bronchial preparations.

**Methods:** Bronchial explants from resected lungs were incubated with roflumilast, roflumilast N-oxide and/or formoterol and then stimulated with LPS. An ELISA was used to measure levels of TNF-α and chemokines in the culture supernatants.

**Results:** At a clinically relevant concentration (1 nM), roflumilast N-oxide and roflumilast consistently reduced the release of TNF-α, CCL2, CCL3, CCL4, CCL5 and CXCL9 (but not CXCL1, CXCL5, CXCL8 and IL-6) from human bronchial explants. Formoterol alone decreased the release of TNF-α, CCL2, and CCL3. The combination of formoterol with roflumilast (1 nM) was more potent than roflumilast alone for inhibiting the LPS-induced release of TNF-α, CCL2, CCL3, CCL4, and CXCL9 by the bronchial explants.

**Conclusions:** At a clinically relevant concentration, roflumilast N-oxide and its parent compound, roflumilast, reduced the LPS-induced production of TNF-α and chemokines involved in monocyte and T-cell recruitment but did not alter the release of chemokines involved in neutrophil recruitment. The combination of formoterol with roflumilast enhanced the individual drugs’ anti-inflammatory effects.

## Background

The ubiquitous second messengers cyclic adenosine monophosphate (cAMP) and cyclic guanosine monophosphate (cGMP) regulate many cell functions. After synthesis by cyclases, their intracellular concentrations are determined by a superfamily of hydrolases, the phosphodiesterases (PDEs). This superfamily comprises more than 21 genes and 11 families of PDEs that differ in their substrate specificity or intracellular location (Zuo et al., 2019). The PDE4 family corresponds to cAMP-specific hydrolyzing enzymes; its members are involved in the release of pro-inflammatory mediators from inflammatory and structural lung cells, and in the regulation of airway smooth muscle tone (Zuo et al., 2019). Therefore, PDE4 inhibitors have been developed to target obstructive inflammatory airway diseases such as asthma and chronic obstructive pulmonary disease (COPD). In these diseases, there is a strong pharmacological and clinical rationale for combining a PDE4I with an inhaled long-acting β_2_-agonist (LABA).

Roflumilast and other PDE4 inhibitors have been shown to reduce allergen- and exercise-induced responses in asthmatic patients (Timmer et al., 2002; Bardin et al., 2015; Singh et al., 2016). A clinical benefit of roflumilast has been demonstrated in patients with mild-to-moderate asthma (Bateman et al., 2015; Meltzer et al., 2015). Roflumilast is also an effective add-on treatment in patients with moderate-to-severe asthma who are inadequately controlled by an inhaled corticosteroid plus a LABA and montelukast (Bateman et al., 2016). In patients with COPD, the add-on administration of roflumilast and other PDE4 inhibitors offers a number of benefits—primarily a lower likelihood of exacerbations (Janjua et al., 2020). Roflumilast is the first PDE4 inhibitor to have been approved by the regulatory authorities for the treatment of severe COPD. The analyses of recent trials and a cost-effectiveness study support the use of roflumilast as a treatment option for severe COPD patients with chronic bronchitis and two or more exacerbations in the prior year despite optimal inhaled therapy (Kiff et al., 2018; Martinez et al., 2018).

The anti-inflammatory effects of oral PDE4 inhibitors (e.g., roflumilast, cilomilast, and BAY 19-8004) or inhaled PDE4 inhibitors (CHF6001) have not been clearly established in COPD patients when assessed as the inflammatory cell count in a bronchial biopsy or the levels of inflammatory biomarkers in induced sputum (Gamble et al., 2003; Grootendorst et al., 2003; Profita et al., 2003; Grootendorst et al., 2007; Wells et al., 2015; Singh et al., 2016; Rabe et al., 2018; Singh et al., 2019). Although roflumilast influences many *in vitro* functions in the human cells involved in COPD (Hatzelmann et al., 2010; Buenestado et al., 2012; Buenestado et al., 2013), the PDE4 inhibitors’ anti-inflammatory effects on human bronchial explants have not previously been assessed.

After oral administration of roflumilast at the clinically recommended dose (500 µg/day), the steady-state free plasma level (∼1-2 nM) of roflumilast-N-oxide (the active metabolite of roflumilast) is maintained over the 24 h dosing interval (Hermann et al., 2007; Lahu et al., 2010). The main objective of the present study was to investigate the effects of roflumilast and roflumilast N-oxide (particularly at the clinically relevant concentration) alone and combined with the LABA formoterol in a human bronchial explant model on the lipopolysaccharide (LPS)-induced release of tumor necrosis factor (TNF)-α, interleukin-6 (IL-6) and a panel of chemokines involved in the pathophysiology of COPD (Barnes, 2009; Barnes, 2018). TNF-α amplifies neutrophilic inflammation and activates macrophages. IL-6 is a pleiotropic cytokine that amplifies inflammation and promotes TH2 cell-mediated and TH17 cell-mediated immunity. The panel of chemokines included chemokines involved in lung infiltration by T-lymphocytes (CCL3 (MIP-1α), CCL4 (MIP-1β), CCL5 (RANTES) and CXCL9 (MIG)), monocytes (CCL2 (MCP-1), CCL3 and CCL4), eosinophils (CCL5), and neutrophils (CXCL1 (GRO-α), CXCL5 (ENA-78) and CXCL8 (IL-8)).

## Methods

### Study Population and Tissue Preparation

The use of resected lung tissues for research purposes was approved by the local institutional review board (CPP IdF VIII, Boulogne-Billancourt, France; reference: DC_2010_1221), and all patients gave their informed consent prior to surgery. Human bronchial explants were obtained from 31 patients (mean ± standard error of the mean (SEM) age: 63 ± 2 years; M:F sex ratio: 26:5, mean ± SEM FEV1: 87 ± 4% predicted); FEV1/FVC ratio: 0.78 ± 0.3; smokers/ex-smokers *n* = 19/*n* = 12 (mean ± SEM pack years: 50 ± 6); and COPD (*n* = 6; FEV1/FVC<0.7; airflow limitation severity: GOLD 1 for 4 patients and GOLD 2 for 2; none were being treated with an inhaled corticosteroid)) undergoing surgical resection for lung carcinoma who had not received preoperative anticancer chemotherapy or radiotherapy. After the removal of connective tissues and adherent lung parenchyma, the bronchial segments (inner diameter: 0.5 to 2 mm) were washed once in complete medium (Roswell Park Memorial Institute 1640 medium, supplemented with 2 mM L-glutamine, 100 µg/ml streptomycin, and 100 IU/ml penicillin) and cut into rings of ∼5–7 mm in length.

### Short-Term Culture of Human Bronchial Explants

The bronchial explants were cultured according to the method described by Morin et al. (Morin et al., 2008). Paired bronchial rings of similar diameter and length were subdivided equally in a 12-well culture plate (∼50 mg tissue per well) containing complete culture medium (2 ml per well) and placed in a humidified 5% CO_2_ incubator at 37°C. After overnight incubation, the culture medium was renewed and the explants were treated for an hour with either roflumilast, roflumilast N-oxide (both 0.1–1000 nM), or vehicle [dimethyl sulfoxide (DMSO), 0.1% final concentration]. Lipopolysaccharide (1 µg/ml) was then added (or not, in non-stimulated preparations) to the culture medium for 24 h. In a different series of experiments, the explants were treated for one hour with roflumilast (1 nM or 100 nM), vehicle, formoterol (10 nM), or roflumilast (1 nM or 100 nM) combined with formoterol (10 nM) prior to LPS stimulation for 24 h. The supernatants were then collected, centrifuged and stored at −80°C prior to cytokine assays. The optimal LPS concentration (1 µg/mL) and incubation time (24 h) were based on our model of human lung parenchymal explants (Buenestado et al., 2013) and were determined from previous experiments (data not shown). The maximal DMSO concentration in culture wells (0.1%) did not affect the LPS-induced cytokine release. All wells were run in duplicate for each series of experiments on samples from a resected lung. Cytotoxicity was assessed by measuring lactate dehydrogenase (LDH) levels in the supernatant after 24 h of culture, using an enzyme assay (Cayman Chemical Europe, Tallinn, Estonia). Neither the vehicle nor any of the compounds used in this study enhanced LDH release.

### Cytokine Assays

The supernatant levels of the selected cytokines were measured in conditioned media obtained after the incubation of bronchial explants in the absence or presence of LPS (1 μg/mL) for 24 h in the series of experiments with roflumilast, its active metabolite roflumilast N-oxide, and formoterol. The cytokine levels were assayed with an ELISA (Duoset Development System), according to the manufacturer’s instructions (R&D Systems Europe, Lille, France). Optical density was determined at 450 nm with an MRX II microplate reader (Dynex Technologies, Saint-Cloud, France). Cytokine concentrations were expressed in ng/100 mg of wet tissue. The assays’ limits of detection were 4 pg/mL for CCL3 and CXCL11, 8 pg/mL for TNF-α, CCL2, CCL5, CXCL5, and CCL4, 10 pg/mL for IL-6, 16 pg/mL for CXCL1, CXCL8, and CXCL10, and 32 pg/mL for CXCL9.

### The Lactate Dehydrogenase Assay

To test the various compounds’ impact on tissue viability, LDH levels in the supernatants after 24 h of tissue culture were determined using a commercially available colorimetric assay, according to the manufacturer’s instructions (Cayman Chemical Europe). As a positive control, bronchial tissues were homogenized on ice using a Bransonic 221 sonicator for 2 min in 10% Triton X100 in phosphate-buffered saline. After centrifugation (15,000 g, 15 min, 4°C), the supernatants were stored at −80°C. The optical density was determined at 490 nm. The assay’s limit of detection was 300 µU/ml.

### Materials

Lipopolysaccharide (from *E. coli* serotype 0111:B4), formoterol fumarate dehydrate, DMSO, acetylcholine and histamine were purchased from Sigma (St. Louis, MO, USA). Roswell Park Memorial Institute 1640 medium, penicillin-streptomycin stabilized solution, and L-glutamine were purchased from Eurobio Biotechnology (Les Ulis, France). Roflumilast and roflumilast-N-oxide were synthesized by Nycomed GmbH (Konstanz, Germany). All other chemicals were of analytical grade and were obtained from Prolabo (Fontenay sous Bois, France). All cell culture plastics were from CCL (Nemours, France). Stock solutions of the PDE4 inhibitors were prepared in DMSO at 10 mM. A 2.5 mM formoterol stock solution was prepared in 10% DMSO. All subsequent dilutions were prepared daily in complete culture medium.

### Statistical Analysis

Data points in graphs and values in the text and figure legends represent the mean ± standard error of the mean (SEM); *n* represents the number of patients from whom bronchial preparations were obtained. Sigmoidal curves were plotted to analyze the effects of roflumilast and roflumilast N-oxide on LPS-induced TNF-α production. Potency data were expressed as the–log EC_50_ (pD2). Statistical analyses were performed on log-transformed data using either paired Student’s t test or a one-way repeated-measures ANOVA followed by Dunnett’s post-test for multiple comparisons. The threshold for statistical significance was set to *p* < 0.05. All analyses were performed using GraphPad Prism^®^ software (version 7.0, GraphPad Software Inc., San Diego, CA, USA).

## Results

### Characterization of the LPS-Induced Response in Human Bronchial Explants

The selection of the cytokines assessed in the present study (TNF-α, CCL2, CCL3, CCL4, CCL5, and CXCL1, CXCL5, CXCL8, CXCL9) was based on (i) our previous experiments on human lung parenchyma explants (Buenestado et al., 2013), and (ii) each cytokine’s known role in the pathophysiology of COPD (Morin et al., 2008; Barnes, 2018). Levels of IL-6 were also assessed in a small number of experiments. The LPS-induced increases in cytokine release from human bronchial explants were much lower than in the previously reported, similar experiments on human parenchymal explants (Buenestado et al., 2013).

### Roflumilast and Its Active Metabolite Roflumilast N-oxide Reduced the Release of TNF-α From LPS-Stimulated Human Bronchial Explants

Unstimulated bronchial explants released a basal amount of TNF-α (mean ± SEM: 208 ± 35 pg/100 mg, *n* = 8). Exposure to LPS caused a 2.9-fold increase in TNF-α release (542 ± 73 pg/100 mg, *p* < 0.01, *n* = 8).

Roflumilast and its N-oxide inhibited the spontaneous release of TNF-α from unstimulated bronchi in a concentration-dependent manner, with pD2 values of 9.6 ± 0.3 (*n* = 8) and 9.4 ± 0.3 (*n* = 8) respectively, and maximal inhibition of 62 ± 7% and 72 ± 6%, respectively.

**FIGURE 1:**
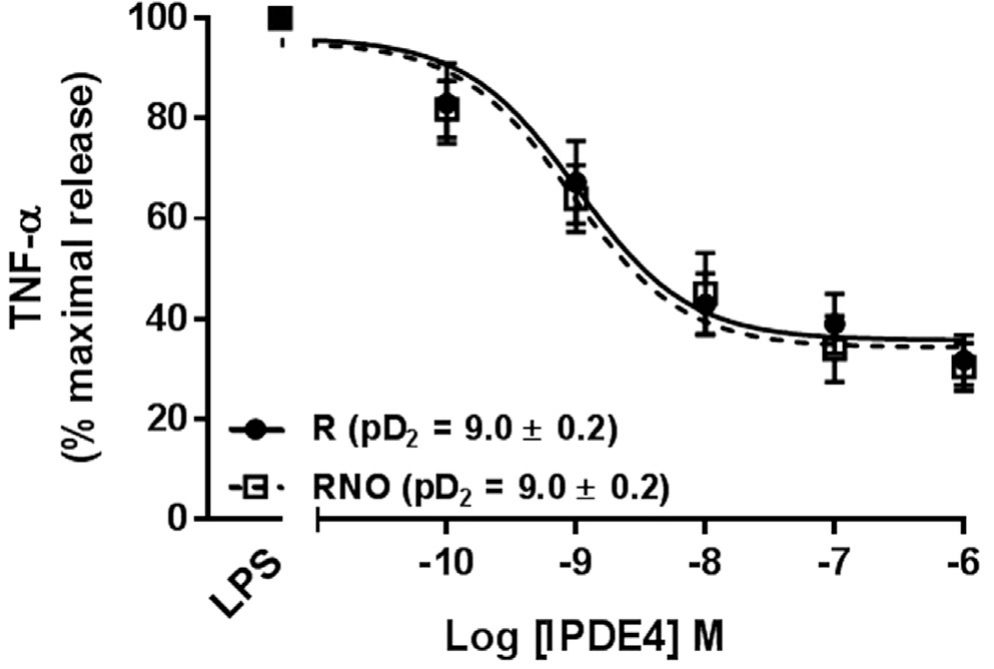
Effects of roflumilast (solid line) and roflumilast N-oxide (dashed line) on the release of TNF-*α* from LPS-stimulated human bronchial explants. The explants were incubated with roflumilast, roflumilast N-oxide (0.1–1,000 nM) or vehicle before addition of LPS (1 µg/mL) for 24 h. The data are quoted as the mean ± SEM of 8 different experiments and are expressed as the percentage of maximum release of TNF-α following LPS and vehicle (0.1% DMSO).

Roflumilast and roflumilast N-oxide (0.1 nM - 1,000 nM) also reduced the LPS-triggered release of TNF-α in a concentration-dependent manner (Figure 1). The maximal inhibition (approximately 68% at 1,000 nM) was similar for the two compounds. The half-maximum inhibition (EC_50_) was obtained at a concentration of ∼1 nM, in line with the compounds’ previously reported potencies for inhibiting PDE4 (Hatzelmann et al., 2010). At 1 nM (a clinically relevant plasma concentration), roflumilast N-oxide and roflumilast reduced the release of TNF-α by 36 ± 7% and 32 ± 5%, respectively (*p* < 0.05); this is again in line with findings in other cellular systems (Hatzelmann et al., 2010), including human lung macrophages and parenchymal explants (Buenestado et al., 2012; Buenestado et al., 2013). Neither the vehicle nor any of the compounds used in the present study were associated with a detectable increase in LDH levels in the bronchial explants’ supernatants.

### Effects of Roflumilast and Its Active Metabolite on the Release of CC and CXC Chemokines From LPS-Stimulated Human Bronchial Explants

Unstimulated bronchial explants produced variable amounts of chemokines (Table 1). The highest levels of baseline release were observed for CCL2, CXCL1 and CXCL8. Addition of LPS resulted in relatively modest increases in chemokine production, with the largest increase being observed for CCL4 (a 2.6-fold increase). The increase in CXCL8 was not significant (Table 1). CXCL10 and CXCL11 were not detected in the supernatants - even after stimulation with LPS.

**TABLE 1:** Chemokine production by paired preparations of LPS-stimulated bronchial explants.

CC chemokines	LPS−	LPS+	Fold increase	CXC chemokines	LPS−	LPS+	Fold increase
CCL2 (*n* = 9)	34.4 ± 7.1	61.5 ± 11.4**	1.8	CXCL1 (*n* = 6)	65.1 ± 13.1	151.1 ± 23.8**	2.3
CCL3 (*n* = 9)	3.4 ± 0.6	7.6 ± 0.9***	2.2	CXCL5 (*n* = 6)	8.8 ± 3.5	16.7 ± 4.9*	1.9
CCL4 (*n* = 10)	3.0 ± 0.6	7.9 ± 1.6**	2.6	CXCL8 (*n* = 11)	457.8 ± 85.2	656.4 ± 107.6	1.4
CCL5 (*n* = 10)	0.10 ± 0.01	0.19 ± 0.03**	1.9	CXCL9 (*n* = 8)	2.0 ± 0.6	4.6 ± 1.3*	2.3

Bronchial explants were treated (or not) with LPS (1 µg/mL). Supernatants were harvested at 24 h and assayed for chemokine levels in an ELISA. Data are expressed in ng/100 mg bronchial explant. The results correspond to the mean of the number of independent experiments given in brackets (n). *p < 0.05; **p < 0.01 and ***p < 0.001 vs. LPS (paired t-test).

In experiments with non-stimulated lung explants, roflumilast (100 nM) inhibited the release of the four CCL chemokines (from an 88% relative reduction for CCL3 to a 40% relative reduction for CCL5) and (to a much lesser extent) CXCL5 and CXCL9 (relative reductions of 23 and 22%, respectively) (Supplementary Table S1). In contrast, 1 nM roflumilast did not significantly inhibit the release of the CXCL chemokines (Supplementary Table S1).

The two PDE4 inhibitors showed very similar effects on the LPS-induced release of each of the investigated chemokines. Complete, selective inhibition of PDE4 (by 100 nM roflumilast and 100 nM roflumilast-N-oxide) consistently but only partially reduced the LPS-induced release of CCL2, 3, 4, and 5, and CXCL9. However, the LPS-induced release of CXCL1 and CXCL5 was unaffected by the PDE4 inhibitors, and that of CXCL8 was slightly and significantly reduced (by 25%) by roflumilast but not significantly by roflumilast-N-oxide (Table 2). This apparent discrepancy might be due to the lower number of experiments with roflumilast N-oxide (6, versus 11 with roflumilast). The degree of inhibition was concentration-dependent for both roflumilast and roflumilast N-oxide (Table 2). At 1 nM (a concentration that corresponds to plasma levels of free roflumilast N-oxide in a therapeutic setting), the active metabolite and roflumilast significantly lowered the release of the four CCL chemokines and CXCL9 (ranging from a 19% relative reduction for CXCL9 to a 64% relative reduction for CCL4) (Table 2).

**TABLE 2:** Effects of roflumilast and roflumilast N-oxide on the release of CC and CXC chemokines by human bronchial explants stimulated with LPS.

Chemokine (ng/100 mg)	LPS (1 µg/mL)					
	*T*	*R* (1 nM)	*R* (100 nM)	*T*	RNO (1 nM)	RNO (100 nM)
CCL2 (*n* = 9/5)	61.5 ± 11.4	45.8 ± 8.6**	35.1 ± 7.6***	51.2 ± 13.4	37.4 ± 7.9*	27.2 ± 7.6*
		25%	43%		27%	47%
CCL3 (*n* = 9/6)	7.6 ± 0.9	4.1 ± 1.0*	1.9 ± 0.4***	8.2 ± 0.7	4.2 ± 0.6*	2.3 ± 0.5**
			75%		49%	72%
CCL4 (*n* = 10/6)	7.9 ± 1.6	4.8 ± 1.5**	2.0 ± 0.7***	6.7 ± 1.2	2.4 ± 0.5***	1.4 ± 0.3***
		39%	74%		64%	79%
CCL5 (*n* = 9/5)	0.19 ± 0.03	0.14 ± 0.03**	0.11 ± 0.01***	0.18 ± 0.1	0.14 ± 0.02*	0.08 ± 0.01**
		26%	42%		22%	55%
CXCLl (*n* = 6/5)	151.1 ± 23.8	116.7 ± 21.6	126.3 ± 32.2	139.5 ± 25.1	115.9 ± 26.4	107.4 ± 28.9
CXCL5 (*n* = 8/4)	16.6 ± 3.7	15.8 ± 3.7	13.7 ± 2.7	19.5 ± 6.9	21.9 ± 6.9	18.9 ± 4.9
CXCL8 (*n* = 11/6)	656.4 ± 107.6	529.6 ± 79.3*	493.7 ± 62.7**	701.6 ± 127.6	713.2 ± 129.2	636.7 ± 133.3
		20%	25%			
CXCL9 (*n* = 13/9)	4.8 ± 0.9	3.6 ± 0.8**	2.7 ± 0.6***	4.6 ± 1.3	3.8 ± 1.1*	2.9 ± 0.9**
		25%	43%		18%	37%

Bronchial explants were pre-treated with vehicle (T), roflumilast (R: 1 nM or 100 nM) or roflumilast N-oxide (RNO: 1 nM or 100 nM) before being stimulated with LPS (1 µg/mL) for 24 h. The data are quoted as the mean ± SEM for the number of independent experiments indicated in brackets for the two compounds (R/RNO). *p < 0.05; **p < 0.01; ***p < 0.001 vs. LPS (T). The significant results are also expressed as the percentage inhibition vs. T (LPS alone).

### Effects of Combining Roflumilast With Formoterol on the LPS-Induced Release of TNF-α, IL-6 and Chemokines by Human Bronchial Explants

Given that roflumilast and roflumilast N-oxide exerted similar inhibitory effects on the LPS-induced release of TNF-α and chemokines, only roflumilast was studied at 1 and 100 nM (giving the maximal inhibitory effect) in the following series of experiments. Since both roflumilast and roflumilast-N-oxide prevent the breakdown of cAMP by PDE4-family enzymes, *in vitro* β_2_-adrenoceptor agonists may further increase intracellular cAMP levels and thereby enhance the anti-inflammatory effects of these PDE4 inhibitors. Therefore, the objective of this series of experiments was to assess the effects of formoterol (alone and in combination with roflumilast) on the LPS-induced release of TNF-α, IL-6 or chemokines. Formoterol was used at 10 nM - a concentration considered to be optimal (Korn et al., 2001; Spoelstra et al., 2002; Salvator et al., 2020; Victoni et al., 2017).

Treatment of bronchial explants with formoterol was associated with significantly lower LPS-induced release of TNF-α, CCL2, CCL3 and IL-6 (by 33%, 27%, 28%, and 25%, respectively; Table 3). Formoterol also reduced the basal production of these cytokines and of CCL4 and CCL5 (Supplementary Table S1).

**TABLE 3:** Effects of roflumilast alone or combined with formoterol on the release of TNF-α, IL-6 and CC and CXC chemokines by human bronchial explants stimulated with LPS.

Cytokine (ng/100 mg)	LPS (1 µg/mL)					
	*T*	*R (1 nM)*	*R (100 nM)*	*F*	*F + R (1 nM)*	*F + R (100 nM)*
TNF-α (*n* = 8)	0.54 ± 0.07	0.32 ± 0.10**	0.14 ± 0.05***	0.36 ± 0.09*	0.20 ± 0.06***	0.09 ± 0.03***
		41%	74%	33%	63%	83%
CCL2 (*n* = 9)	61.5 ± 11.4	45.8 ± 8.6**	35.1 ± 7.6***	44.9 ± 8.6*	35.6 ± 7.7***	27.9 ± 6.4***
		25%	43%	27%	42%	55%
CCL3 (*n* = 9)	7.6 ± 0.9	4.1 ± 1.0*	1.9 ± 0.4***	5.5 ± 0.8*	2.8 ± 0.6***	1.5 ± 0.3***
		46%	75%	27%	63%	80%
CCL4 (*n* = 10)	7.9 ± 1.6	4.8 ± 1.5**	2.0 ± 0.7***	6.7 ± 1.5	3.3 ± 1.0***	1.6 ± 0.6***
		39%	74%	15%	58%	79%
CCL5 (*n* = 9)	0.19 ± 0.02	0.14 ± 0.03**	0.11 ± 0.01**	0.15 ± 0.03	0.11 ± 0.01**	0.09 ± 0.01***
		26%	42%	21%	42%	53%
CXCLl (*n* = 6)	151.1 ± 23.8	116.7 ± 21.6	126.3 ± 32.2	119.6 ± 22.7	134.5 ± 42.5	105.5 ± 35.9
CXCL5 (*n* = 6)	16.6 ± 3.7	15.8 ± 3.7	13.7 ± 2.7	16.0 ± 4.0	15.9 ± 3.2	13.2 ± 1.9
CXCL8 (*n* = 11)	656.4 ± 106.7	529.6 ± 79.3*	477.8 ± 62.7**	613.8 ± 97.1	531.5 ± 80.3	434.9 ± 80.2**
		20%	25%	6%	19%	34%
CXCL9 (*n* = 13)	4.8 ± 0.9	3.6 ± 0.8**	2.7 ± 0.6***	4.3 ± 0.9	2.9 ± 0.6***	2.3 ± 0.5***
		25%	43%	10%	39%	48%
IL-6 (*n* = 9)	373.2 ± 78.3	323.8 ± 85.6	241.2 ± 44.1**	281.4 ± 52.2*	284.2 ± 69.8	208.2 ± 59.3***
			35%	25%		44%

Bronchial explants were pre-treated with vehicle (T), roflumilast (R: 1 nM or 100 nM), formoterol (F: 10 nM) or a combination of formoterol and roflumilast (F + R) before being stimulated with LPS (1 µg/mL) for 24 h. Data are quoted as the mean ± SEM for the number of independent experiments indicated in brackets. *p < 0.05; **p < 0.01; ***p < 0.001 vs. LPS (T). The significant or relevant results (in italics) are also expressed as the percentage inhibition vs T (LPS alone).

**FIGURE 2:**
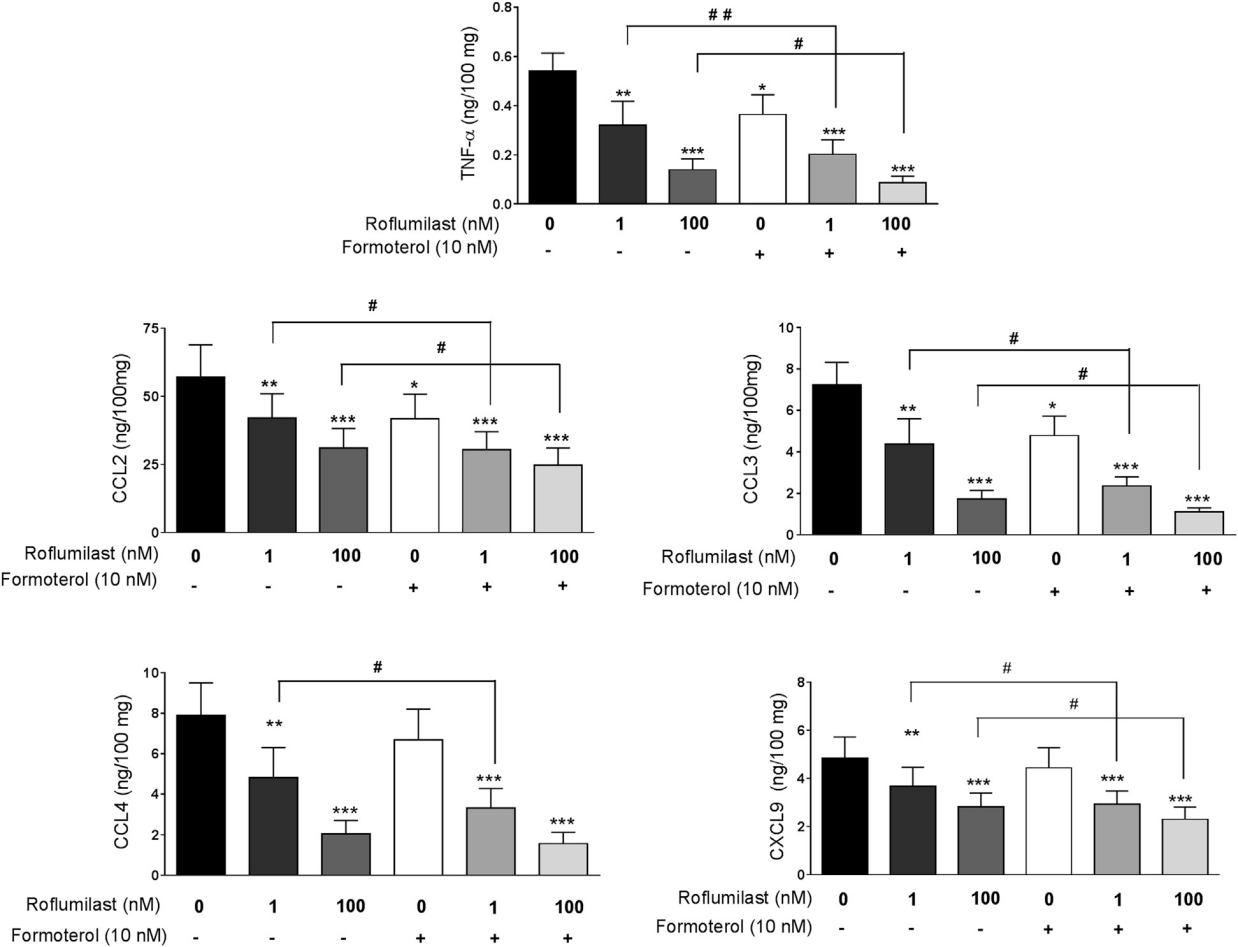
Effects of formoterol alone and combined with roflumilast on LPS-stimulated release of TNF-α, CCL2, CCL3, CCL4 and CXCL9 by human bronchial explants. The explants were incubated with roflumilast (1 and 100 nM), formoterol (10 nM) or vehicle before stimulation with LPS (1 µg/mL) for 24 h. The data are quoted as the mean ± SEM of 7 to 9 different experiments. *p < 0.05, **p < 0.01, ***p < 0.001 vs. LPS, #*p* < 0.05 and ##*p* < 0.01.

Concomitant incubation with formoterol (10 nM) and roflumilast (1 nM) was associated with significantly greater inhibition (relative to roflumilast alone) of the release of TNF-α, three of the four CCL chemokines (CCL2,3,4), and CXCL9 (Table 3, Figure 2). For example, the LPS-induced release of TNF-α and CCL2 was further reduced by 37% and by 23% with the addition of formoterol, relative to 1 nM roflumilast alone. We used the Abbott formula (Gisi, 1996) [%expected = %inhibition roflumilast (R) + %inhibition formoterol (F) – (R*F/100)] to estimate the extent of the interaction between roflumilast and formoterol. The ratio between the observed efficacy of the combination of roflumilast and formoterol and the expected efficacy calculated with the Abbott formula was very close to 1 - suggesting an additive effect rather than a synergistic effect. For the cytokines inhibited by roflumilast, the mean [95%CI] ratio was 0.95 (0.83–1.07) and 1.01 (0.87–1.14) in nonstimulated and LPS-stimulated explants, respectively. In contrast, the LPS-induced or basal release of the CXCL chemokines and IL-6 was not affected by exposure to formoterol (Table 3), and the weak and mostly non-significant inhibitory effects of roflumilast at 100 nM were not significantly enhanced by formoterol.

## Discussion

In the present study, we evaluated the potential anti-inflammatory properties of roflumilast and its active metabolite alone or in combination with a LABA in an *in vitro* model of the human bronchi. Our results demonstrated for the first time that at clinically relevant concentrations, (i) the PDE4 inhibitors roflumilast N-oxide and roflumilast reduce the release of TNF-α and of some chemokines CCL2, CCL3, CCL4, CXCL9 (but not CXCL1, CXCL5 or CXCL8) from LPS-stimulated human bronchial explants, and (ii) the combination of formoterol with roflumilast is more potent than each component alone for inhibiting the LPS-induced release of TNF-α and chemokines by the bronchial explants.

Here, we employed a bronchial explant model to establish whether the acute inflammatory response triggered by LPS was inhibited by roflumilast, roflumilast N-oxide, and formoterol. We selected a clinically relevant concentration of endotoxin (1 µg/mL), based on those measured in the bronchoalveolar fluid (BALF) of patients with acute respiratory distress syndrome or pneumonia and considering a 100-fold dilution of BALF-endotoxin concentrations (Miller et al., 1992; Martin et al., 1997; Flanagan et al., 2001; Nys et al., 2003). A previous study of human lung parenchyma explants (Buenestado et al., 2013) (i) featured greater LPS-induced increases in cytokine levels (using the same assays as in the present study of human bronchial explants) in most cases, and (ii) highlighted the release of cytokines (such as CXCL8 and CXCL10) that were either not detected or not released in response to LPS in the present study. These differences probably reflect differences in cell populations in bronchial and parenchymal explants (e.g., high numbers of inflammatory cells such as macrophages in the parenchymal tissue) as also suggested by the weaker release of leukotrienes and histamine by human bronchial explants when compared to lung parenchyma (Salari et al., 1985). However, exposure of the bronchial explants to LPS caused significant relative increases in the level of many cytokines involved in the pathophysiology of chronic obstructive respiratory diseases. Although this model usefully features the full set of bronchial cells in their normal ratios and spatial configuration, the epithelial layer’s barrier properties are compromised by exposure to challenge agents. Further, the effect of the circulation is absent. However, this model enabled us to explore the anti-inflammatory properties of roflumilast and its active metabolite under conditions that more closely resemble those seen in intact lung than *in vitro* cell culture models; the latter provide information on the responses of a single, isolated cell type only.

The PDE4s are the most active cAMP-hydrolyzing enzymes in the human bronchus (de Boer et al., 1992; Cortijo et al., 1993; Torphy et al., 1993; Yougbare et al., 2011). Among the PDE4 isoenzymes present in airways, the subtypes A, C and D are present in human epithelial cells (Fuhrmann et al., 1999), the subtypes A and B are highly detected in the inflammatory cells (Zuo et al., 2019), and the subtype D is the most prominent PDE4 within airway smooth muscle (Billington et al., 2008). Roflumilast and its active metabolite inhibit all the PDE4 isoenzymes, with IC_50_ values in the low nanomolar range (with the exception of PDE4C, which is inhibited with a slightly lower potency) (Hatzelmann et al., 2010). After oral administration of roflumilast at the clinical recommended dose, the steady-state free fraction of roflumilast-N-oxide in plasma (1–2 nM) is maintained over the 24 h dosing interval (Hermann et al., 2007; Lahu et al., 2010). The distribution volume of roflumilast-N-oxide is relatively low and the concentrations in lung tissue are probably similar to the plasma concentrations (Hermann et al., 2007; Lahu et al., 2010). At a clinically relevant concentration (1 nM), the active metabolite and its parent compound exerted similar effects on the production of the selected set of cytokines. Furthermore, roflumilast N-oxide and roflumilast’s respective potencies for suppressing TNF-α release from LPS-stimulated human bronchial explants was quite similar to their potencies for inhibiting PDE4 (Hatzelmann et al., 2010).

The mechanisms of the inhibitory effect of the PDE4 inhibitors on the LPS-induced cytokine’s production have not been explored in the present work on human bronchial explants, notably because such a complex system with mutually interacting cells expressing different cAMP-hydrolyzing PDEs and different inflammatory pathways make the exploration of mechanisms much difficult. Furthermore, it is well known that PDE4 inhibitors act by enhancing levels of cAMP, activating the cAMP acceptors protein kinase A and Epac, and inhibiting the transcription of inflammatory genes. It has previously been reported that the production of TNF-α, IL-6, CCL2, CCL3, CCL4, CCL5 and CXCL9 is regulated by cAMP-dependent pathways (modulated by adenosine, prostaglandin E_2_, β_2_-adrenoceptor agonists, and PDE4 inhibitors) in human monocytes, lung macrophages, airway smooth muscle cells, epithelial cells, and lung parenchymal explants (Bryn et al., 1950; Hallsworth et al., 2001; Takayama et al., 2002; Buenestado et al., 2010; Buenestado et al., 2013; Van Ly et al., 2013; Wyatt et al., 2014; Wex et al., 2015). In contrast, the PDE4 inhibitors did not influence or only weakly influenced the release of the neutrophil attractants CXCL1, CXCL5 and CXCL8. This latter finding is in line with our previous studies of LPS-stimulated human lung explants and macrophages, in which an A_2A_ adenosine receptor agonist, an adenylyl cyclase activator (forskolin), a β_2_-adrenoceptor agonist (formoterol), and PDE4 inhibitors, curbed the release of TNF-α, CCL2, CCL3, CCL4, CXCL9, and CXCL10 but not CXCL1, CXCL5 and CXCL8 (Buenestado et al., 2010; Buenestado et al., 2012; Buenestado et al., 2013). In other *in vitro* studies, CXCL1 and/or CXCL8 were unaffected or only weakly affected or by agents that increase cAMP intracellular concentrations in human monocytes/macrophages, human lung fibroblasts, airway smooth muscle cells, and bronchial epithelial cells (Yoshimura et al., 1997; Dent et al., 1998; Hallsworth et al., 2001; Korn et al., 2001; Spoelstra et al., 2002; Donnelly et al., 2010). Furthermore, a high concentration (1 µM) of cilomilast reduced TNF-α release by sputum cells from COPD patients, whereas CXCL8 release was not significantly inhibited (Profita et al., 2003). Taken as a whole, these literature data and the results of the present study suggest that the production of CXCL1, CXCL5 and CXCL8 in many human pulmonary cells and lung tissues is rather insensitive to cAMP modulators.

In the present study, formoterol exerted the same overall activity on the selected cytokines, i.e. partial inhibition of the release of TNF-α, CCL2, CCL3, and CCL5. In other preparations (such as human airway epithelial cells (Holden et al., 2010), smooth muscle cells (Kaur et al., 2008), monocyte derived-macrophages (Donnelly et al., 2010), human lung fibroblasts (Spoelstra et al., 2002) and lung parenchymal explants (Buenestado et al., 2013)), formoterol (10 nM) was also shown to reduce cytokine production. The combination of formoterol with roflumilast exerted an additive effect enhancing the roflumilast’s anti-inflammatory effects (in a range of 20–25%) on bronchial explants. A synergistic effect has been reported in human eosinophils and neutrophils; however, PDE4 accounts for virtually all the cAMP-metabolizing capacity in these cell types (Buenestado et al., 2013). Only additive effects have been observed with either β2-adrenoceptor agonists or other adenylyl cyclase stimulants in other human inflammatory cells such as the blood monocytes or the pulmonary macrophages which express other cAMP-hydrolyzing PDEs (Buenestado et al., 2013). In bronchial explants with interacting cells expressing different cAMP-hydrolyzing PDEs, a synergistic effect was unlikely (Buenestado et al., 2013).

Our results did not identify the exact source of the cytokines released in response to LPS or quantify the various bronchial cells’ contributions to roflumilast’s inhibitory effects. In human bronchi, PDE4 controls ∼50% of cAMP hydrolytic activity and the PDE4 enzymes are expressed in all cell types in the lungs, where they exert a predominant hydrolytic activity on cAMP (Yougbare et al., 2011). However, other PDEs (such as PDE3 or PDE1) also contribute to the control of intracellular cAMP levels in some cells (Torphy et al., 1993). The co-expression of PDEs involved in cAMP degradation other than PDE4 might account for the partial reduction in LPS-induced cytokine release from human bronchial explants observed when the PDE4s were fully blocked with 100 nM roflumilast or N-oxide roflumilast (as illustrated in Figure 1). Since formoterol did not inhibit LPS-stimulated cytokine release from human lung macrophages (Victoni et al., 2017), this inflammatory cell type is probably not involved in the formoterol’s additive effect on the reduction in LPS-induced cytokine release by bronchial explants.

In both human parenchymal explants (Buenestado et al., 2013) and bronchial explants (the present study), the production of chemokines (CXCL1, CXCL5 and CXCL8) involved in the recruitment of neutrophils was not inhibited by roflumilast (alone or combined with formoterol) or its active metabolite. However, treatment with roflumilast in some clinical studies reportedly reduced (i) neutrophil accumulation in the BALF following bronchial LPS challenge in human volunteers (Hohlfeld et al., 2008) and (ii) the sputum neutrophil count in COPD patients (Grootendorst et al., 2007). This effect might be related to a direct suppressive effect of roflumilast on blood-derived neutrophil migration (Dunne et al., 2019), by reducing the adherence of resting neutrophils to activated endothelial cells (Sanz et al., 2007) and by inhibiting the self-propagating acetyl-proline-glycine-proline pathway (Wells et al., 2015). A recent clinical study of the treatment of severe COPD patients with an inhaled PDE4 inhibitor (CHF6001) as an add-on to triple inhaled therapy (formoterol combined with a long-acting muscarinic antagonist and a corticoid) showed a reduction in the levels of expression of genes associated with the pathophysiology of COPD (e.g. TNF- α, CCL2,3,4,5 and CXCL9) (Govoni et al., 2020) and in the sputum concentrations of TNF-α, CCL2, CCL4 and CXCL8 but did not evidence a reduction in the neutrophil count (Singh et al., 2019). The reduction in CXCL8 was probably due to the high sputum concentration (∼10 µM) of this inhaled PDE4 inhibitor (Singh et al., 2019). However, a 4-week treatment with roflumilast was shown to reduce the sputum level of CXCL8 and the sputum neutrophil count in a small group of COPD patients treated with short-acting bronchodilators (Grootendorst et al., 2007). This reduction in CXCL8 might also result from a progressive anti-inflammatory action of roflumilast on the bronchial mucosa. In contrast to the latter study, a recent study in a larger population of COPD patients revealed a consistent reduction in the eosinophil counts (but not the neutrophil count) in both bronchial biopsy and sputum samples after 16 weeks of treatment with roflumilast - particularly when the drug was combined with a LABA (Rabe et al., 2018). The inhaled PDE4 inhibitor (CHF6001) was also shown to reduce sputum eosinophil counts in COPD patients with a higher sputum eosinophil count (≥3%) (Singh et al., 2020). This reduction in the eosinophil count might be due (at least in part) to the inhibition of CCL5 release in bronchial explants (as seen in the present study), which might contribute to the reduction in moderate-to-severe exacerbations of COPD (Martinez et al., 2018).

## Conclusions

The present study of human bronchus samples expands the literature observations and demonstrates that roflumilast and its N-oxide metabolite (i) are anti-inflammatory agents and (ii) provide additive anti-inflammatory effects at clinically relevant concentrations when administered concomitantly with formoterol. Our observations supports the results of randomized clinical trials, which suggested that the combination of roflumilast with a LABA provides additional benefit for the prevention of COPD exacerbations (Bateman et al., 2011; Mills et al., 2011; Hanania et al., 2014; Martinez et al., 2018).

The improved clinical efficacy of oral PDE4 inhibitors is thought to be related either to a better tolerability profile (i.e. an increase in the daily dose and the achievement of plasma concentrations that completely block PDE4) or the inhaled administration route (increasing lung concentrations and reducing systemic exposure) (Singh et al., 2019) with compounds with higher *in vitro* potency such as CHF6001 (Lea et al., 2019).

## Data Availability Statement

The dataset supporting the conclusions of this article is available on request from corresponding author.

## Ethics Statement

The studies involving human participants were reviewed and approved by Comité de Protection des Personnes (CPP) IdF VIII, Boulogne-Billancourt, France;. The patients/participants provided their written informed consent to participate in this study.

## Author Contributions

The study was conceived and designed by PD, HS, AB, EN and SGD. AB, TV, EN, EL, and MB contributed to the experiments and data acquisition, with HT overseeing study conduct. The data were analysed by PD, MB, HS, and AB, and all authors revised the manuscript for intellectual content and approved its publication. PD drafted the manuscript.

## Funding

The authors declare that this study received funding from Nycomed/Takeda. The funder was not involved in the study design, collection, analysis, interpretation of data, the writing of this article or the decision to submit it for publication.

## Conflict of Interest

PD has received consulting fees from Astra Zeneca, Boehringer-Ingelheim, Chiesi, GlaxoSmithKline, Menarini, Mylan, Novartis, and Sanofi. HT is an employee of Topadur. SGD has received a grant from AZ for a clinical trial out of the scope of the present study.

The remaining authors declare that the research was conducted in the absence of any commercial or financial relationships that could be construed as a potential conflict of interest.

## Abbreviations

**Abbreviations**: cAMP, cyclic adenosine monophosphate; BALF, Bronchoalveolar fluid; cGMP, cyclic Guanosine Monophosphate; DMSO, Dimethyl Sulfoxide; COPD, Chronic Obstructive Pulmonary Disease; FEV1, Forced Expiratory Volume in 1 s; FVC, Forced Vital Capacity; LABA, Long-acting β2-agonist; PDE4, Phosphodiesterases 4.
