# Supplementary material for: Clinical Relevance of the Anti-inflammatory Effects of Roflumilast on Human Bronchus: Potentiation by a Long-Acting Beta-2-Agonist
Source: Front Pharmacol. 2020 Dec 8;11:598702. doi: 10.3389/fphar.2020.598702 (PMC7754640; doi:10.3389/fphar.2020.598702)
Supplement: Supplementary file 1 [file table1.docx]

Table S1. Effects of roflumilast alone or combined with formoterol on the release of TNF-α and CC and CXC chemokines by unstimulated human bronchial explants.

| Cytokine  (ng/100 mg) | | *T* | | *R (1 nM)* | | *R (100 nM)* | | *F* | | *F + R (1 nM)* | | *F + R (100 nM)* |
| --- | --- | --- | --- | --- | --- | --- | --- | --- | --- | --- | --- | --- |
| TNF-α (n=7) | 0.24 ± 0.04 | | 0.10 ± 0.03**  58% | | 0.07 ± 0.03***  71% | | 0.16 ± 0.05*  33% | | 0.09 ± 0.03***  62% | | 0.05 ± 0.02***  79% | |
| CCL2 (n=7) | 51.6 ± 16.3 | | 34.8 ± 8.9*  32% | | 19.2 ± 3.5*  63% | | 35.7 ± 10.4*  31% | | 23.2 ± 6.2**  55% | | 15.7 ± 3.0**  70% | |
| CCL3 (n=9) | 3.4 ± 0.6 | | 1.3 ± 0.3**  62% | | 0.4 ± 0.1**  88% | | 2.2 ± 0.4*  35% | | 1.1 ± 0.2**  68% | | 0.4 ± 0.1***  88% | |
| CCL4 (n=8) | 2.8 ± 0.5 | | 1.3 ± 0.3**  53% | | 0.5 ± 0.1**  82% | | 1.9 ± 0.5*  32% | | 1.2 ± 0.3*  57% | | 0.4 ± 0.1**  86% | |
| CCL5 (n=7) | 0.12 ± 0.03 | | 0.10 ± 0.03  *17%* | | 0.06 ± 0.02***  50% | | 0.07 ± 0.03*  41% | | 0.09 ± 0.03**  25% | | 0.06 ± 0.03***  50% | |
| CXCLl (n=8) | 90.7 ± 21.6 | | 72.6 ± 14.4 | | 77.7 ± 16.9 | | 55.6 ± 10.7 | | 78.0 ± 23.9 | | 58.6 ± 12.4 | |
| CXCL5 (n=8) | 10.9 ± 2.0 | | 10.4 ± 2.6 | | 8.3 ± 1.8*  24% | | 7.8 ± 1.4  *28%* | | 9.8 ± 2.5 | | 6.1 ± 1.1**  44% | |
| CXCL8 (n=7) | 377.2 ± 45.6 | | 317.4 ± 28.2 | | 332.5 ± 49.5  *12%* | | 370.7 ± 53.4  *2%* | | 320.6 ± 43.3 | | 284.2 ± 36.1*  25% | |
| CXCL9 (n=8) | 3.9 ± 0.5 | | 3.5 ± 0.4 | | 3.1 ± 0.5*  20% | | 2.7 ± 0.5  *31%* | | 3.0 ± 0.7 | | 2.5 ± 0.6*  36% | |

Bronchial explants were pre-treated with vehicle (T), roflumilast (R: 1 nM or 100 nM), formoterol (F: 10 nM) or the combination of roflumilast and formoterol (F+R) without being exposed to LPS for 24 h. The data are quoted as the mean ± SEM for the number of independent experiments indicated in brackets. *p<0.05; **p<0.01; ***p<0.001 vs. LPS (T). The significant or useful (in italics) results are also expressed as percentage inhibitions vs vehicle (T).
